# Exposure to 1‐bromopropane vapors during pregnancy enhances the development of hippocampal neuronal excitability in rat pups during lactation

**DOI:** 10.1002/1348-9585.12135

**Published:** 2020-07-26

**Authors:** Yukiko Fueta, Susumu Ueno, Toru Ishidao, Yasuhiro Yoshida, Yasunari Kanda, Hajime Hori

**Affiliations:** ^1^ Department of Environmental Management and Control School of Health Sciences University of Occupational and Environmental Health Kitakyushu Japan; ^2^ Department of Pharmacology School of Medicine University of Occupational and Environmental Health Kitakyushu Japan; ^3^ Department of Immunology and Parasitology School of Medicine University of Occupational and Environmental Health Kitakyushu Japan; ^4^ Division of Pharmacology National Institute of Health Sciences Kawasaki Japan

**Keywords:** 1‐bromopropane, CA1 field of hippocampus, electrophysiology, postnatal development, toxicology

## Abstract

**Objectives:**

Although 1‐Bromopropane (1‐BP) exposure has been reported to cause neurotoxicity in adult humans and animals, its effects on the development of the central nervous system remain unclear. Recently, we reported delayed developmental neurotoxicity (DNT) upon 1‐BP exposure in rats. Here we aimed to study the effect of prenatal 1‐BP exposure on the hippocampal excitability in the juvenile offspring.

**Methods:**

Pregnant Wistar rats were exposed to vaporized 1‐BP for 20 days (6 h/d) with concentrations of 0 (control), 400, or 700 ppm. Hippocampal slices were prepared from male offspring during postnatal days (PNDs) 13, 14, and 15. Field excitatory postsynaptic potential (fEPSP) and population spike (PS) were recorded simultaneously from the CA1 region.

**Results:**

In the exposed groups, the stimulation/response relationships of fEPSP slope and PS amplitude were enhanced more than in the control group at PND 14. Analysis of fEPSP‐spike coupling demonstrated increased values of Top and Eslope50 in the exposed groups. Real‐time PCR analysis showed a significant increase in the mRNA levels of the adult type Na_v_1.1 Na^+^ channel subunit and the GluR1 glutamate receptor subunit in the hippocampus of the 700 ppm group at PND 14.

**Conclusions:**

Our results provide evidence that prenatal exposure to 1‐BP accelerates developmental enhancement of hippocampal excitability in the pups before eye‐opening. The current study suggests that our evaluation method of DNT is applicable to the industrial chemical 1‐BP.

## 1 INTRODUCTION

1‐Bromopropane (CH_3_‐CH_2_‐CH_2_Br; 1‐BP) is used in several manufacturing processes, including immersion degreasing operations to clean metal, precision instruments, electronics, optical instruments, and ceramics, in addition to being used as a solvent for aerosol applied adhesives in cushion manufacturing industries. The American Conference of Governmental Industrial Hygienists (ACGIH) has describe the potential for 1‐BP to contribute to neurotoxicity, hepatotoxicity, and reproductive and developmental toxicity.^1^, ^2^ A recent critical issue in neurotoxicology is the developmental effects of environmental and occupational chemical substances on the central nervous system (CNS).^3^, ^4^, ^5^ A few industrial chemicals, such as lead, methylmercury, polychlorinated biphenyl, arsenic, and toluene, have been reviewed as causative substances of developmental disorders and sub‐clinical dysfunction of the human CNS.^6^ Approximately 200 industrial chemicals are known to have neurotoxic effects on adult humans, and additional chemicals reported to be neurotoxic in laboratory animals.^6^ However, developmental neurotoxic effects caused by many chemicals, including 1‐BP, remain uncharacterized.

We previously reported that prenatal 1‐BP exposure alters the incidence of kainate‐induced wet‐dog shake behaviors at postnatal day (PND) 14 in pups,^7^ and that it causes delayed adverse effects on the hippocampus after growth.^8^ These results suggest that prenatal 1‐BP exposure has a potential of changing excitability in the CNS after growth, as well as in juvenile rats. Recently, we demonstrated changes in hippocampal excitability, coincident with the eye‐opening period, as a useful ex vivo evaluation of developmental neurotoxicity (DNT) in pups that underwent prenatal administration of valproic acid (VPA).^9^ Prenatal VPA exposure potentiated developmental enhancement of the hippocampal excitability in rat pups at PND 14 and 15, before the eye‐opening day. Here we extended the application of our evaluation method to 1‐BP, and aimed to elucidate whether deviations in hippocampal excitability before eye‐opening were induced by prenatal exposure to 1‐BP vapor in the pups. Hippocampal excitability was investigated via analysis of the stimulation‐evoked field potentials, such as field excitatory postsynaptic potential (fEPSP) and simultaneously recorded population spike (PS) in the same slice. As an excitatory input, fEPSP is recorded from a synaptic area in the CA1 region, where synaptic inputs to the CA1 neurons are localized. PS is recorded from the cell layer of the CA1 region; therefore, it represents output from the CA1 neurons. Thus, the relationship between fEPSPs and PSs can be physiologically construed as input and output of the neurons, respectively. This input/output relationship is represented as fEPSP (EPSP)/spike coupling (E‐S coupling).^10^, ^11^, ^12^ Analysis of E‐S coupling has been used to investigate changes in the neuronal excitability induced by any fluctuation in physiological conditions,^11^, ^12^, ^13^, ^14^, ^15^, ^16^, ^17^ by chemicals,^18^, ^19^, ^20^ or by pathological conditions.^10^, ^21^ We attempted to address DNT by investigating the E‐S coupling in a quantitative fashion.

## 2 MATERIALS AND METHODS

### 2.1 Animals

Thirty male (9‐11 weeks of age) and 64 female (9 weeks of age) Wistar rats were purchased from Kyudo Co., Ltd. or CLEA Japan Inc. All rats were bred in polycarbonate cages. Female rats at a proestrus stage were selected using an impedance checker (MK‐10B, Muromachi Kikai Co. LTD.). One female rat was caged with 1 male rat, and mating was confirmed by the presence of sperm in the vaginal smear or vaginal plug [gestation day (GD) 0]. Sperm‐ or plug‐positive females were then allocated into either the control (0 ppm) or exposed (400 and 700 ppm) groups

The dams were checked for the onset of birth at 9 am, 2 pm, and 6 pm, and the time of each birth was noted. The expected day of delivery, that is, GD 21, was designated as PND 0 for the pups. Any abnormalities in the delivery were checked at PND 1. On PND 2, the litters were standardized to 8 or 10 offspring (eg, 4 males and 4 females, or 5 males and 5 females) by removing any excess offspring after the evaluation of anogenital distance of all pups. General postnatal growth was monitored for ear unfolding, eye opening, testicular descent, and vaginal opening. The body weight of control, 400 and 700 ppm pups was monitored at PNDs 2, 7, 14, 18, 21, and 27 respectively. The experiments were performed under the guidelines of the Ethics Committee of Animal Care and Experimentation in accordance with The Guiding Principle for Animal Care Experimentation, University of Occupational and Environmental Health, Japan, and the Japanese Law for Animal Welfare and Care. Registration No. was AE03‐065.

### 2.2 1‐BP inhalation and breeding conditions

**Figure 1:**
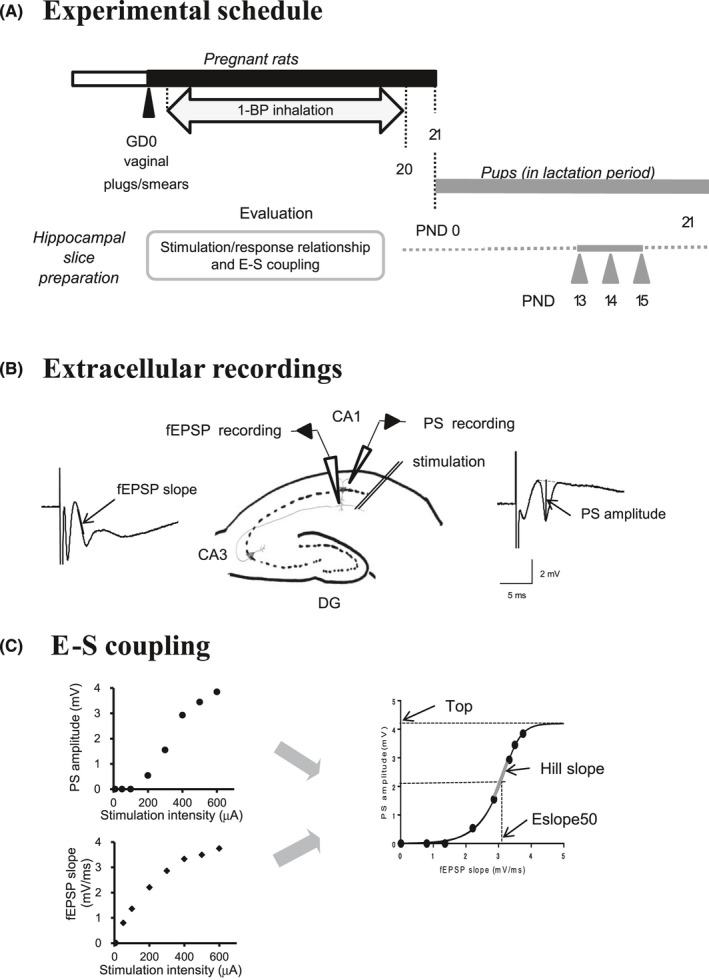
Experimental protocol, scheme of extracellular recordings, and analysis of fEPSP‐Spike (E‐S) coupling. (A) Experimental schedule of inhalation and slice preparation. Dams were exposed to 1‐bromopropane (1‐BP) from gestation days (GDs) 1‐20. Hippocampal slices were prepared at PNDs 13‐15. For evaluation of effects of prenatal 1‐BP exposure, stimulation intensity dependent changes in responses were analyzed in the hippocampal slices, and relationship between the field excitatory postsynaptic potentials (fEPSP) and the population spike (PS), so called fEPSP‐Spike (E‐S) coupling, was investigated. (B) Two extracellular recording electrodes for PS and fEPSP, and bipolar stimulation electrode were set in the CA1 region of the hippocampal slice. The fEPSP and PS recording electrodes were set in the stratum radiatum and near the cell layer following previous study, respectively (Fueta et al, 2018b). Bipolar stimulation electrode supplied electrical stimulation to Schaffer collateral and commissural fibers, which gave synaptic input to the dendrites of CA1 neurons. The distance between the stimulating electrode and the fEPSP recording electrode was measured under a microscope in the recorded hippocampal slice, and was set between 200 and 250 µm in all slices tested. Dashed line represents the cell layers of CA1, CA3, and dentate gyrus (DG) of the hippocampal formation. Closed triangle represents an amplifier. Traces represent typical fEPSP (left) and PS (right) evoked in the CA1 region obtained from a control pup at PND 15 with a stimulation intensity of 600 µA. The thick lines represent the measurements of fEPSP slope and PS amplitude, respectively. (C) E‐S coupling curve created from the stimulation/response (S/R) relationship of fEPSP slope (left below chart) and that of PS amplitude (left above chart) in each slice. The representative data were obtained from the slice of a PND 15 pup from the 400 ppm group. The abscissa axis of E‐S coupling chart represents fEPSP slope, and the vertical axis represents PS amplitude. When S/R relationship in each slice fitted to a logistic curve, the slice data of S/R relationship were provided to create the E‐S coupling curve of the group. Parameters of Top, Eslope50, and Hill slope were calculated from the curve of each slice and are summarized in Table 3

1‐BP was obtained from Kanto Chemical Co., Ltd. The exposure groups were placed in a stainless‐steel inhalation chamber and were exposed to 1‐BP concentrations of 400 or 700 ppm, while the control group (0 ppm) was exposed to filtered room air in the same type of chamber. The concentration of 400 ppm was the lowest observed adverse effect level (LOAEL) obtained from our neurotoxic study of 12‐week repetitive inhalation.^22^ The temperature of the chamber was maintained at 22 ± 1°C with a light period from 7 AM to 7 PM. The exposures were performed for 6 hours per day, between 9 am and 3 pm, for 20 days. The experimental schedule is shown in the Figure 1A. Inhalation breeding cages were made from clear acrylic resin with small holes to avoid stagnation of 1‐BP vapor inside the cage. Breeding conditions of water, food, and chip bedding were maintained in accordance with a previously described method.^8^, ^9^

### 2.3 Electrophysiological recordings and analysis

Previously,^9^ we measured fEPSP and PS evoked with stimulation in the CA1 of hippocampal slices obtained from pups at PNDs 13‐18, and demonstrated an increased developmental enhancement in hippocampal excitability at PNDs 14 and 15 upon prenatal exposure to VPA. The second postnatal week is thought to correspond to the critical period of activity‐dependent synaptogenesis in rats.^23^ Therefore, in the current study, we focused on pups at the age of PNDs 13‐15. For electrophysiological examinations, a pup was separated from its mother immediately before anesthesia without disturbing the suckling. Male pups were selected at random and allocated to the slice study. The total numbers of pups were 17 from the control, 6 from the 400 ppm, and 12 from the 700 ppm groups. Hippocampal slices were prepared as previously described.^9^ PS and fEPSP were recorded in the CA1 region in each slice (Figure 1B). The hippocampal excitability was evaluated via stimulation/response (S/R) relationships of the fEPSP slope and PS amplitude. To study the S/R relationship, stimulation intensity was increased from 20, 50, and 100 µA up to 600 µA in 100 µA increments. Stimulation and recording system used were as described in our previous study.^9^ The PS amplitude and the slope of fEPSP were measured as previously described (Figure 1B).^9^ Plots of PS amplitude vs fEPSP slope were obtained with increasing stimuli in the range of 20‐600 µA, which covered the intensity range from no PS to almost maximal PS amplitude size (Figure 1C). Identical stimulus protocols were applied to each slice. Plotted data were fit to a logistic curve with a built‐in function in GraphPad Prism 6 (GraphPad Software). Three parameters of “Top,” “Eslope50,” and “Hill slope” of the 1‐BP‐exposed groups were compared to those of the control group. In adult slices, maximal PS amplitude was obtained from apparent plateaus of maximal PS.^17^, ^20^ In slices obtained from juvenile rats, we occasionally observed plateaus of submaximal PS, but not of maximal PS, at an intensity of 600 µA. Stimulation intensity of 600 µA with 100‐µs duration was set as the maximum‐dose intensity to avoid the risk of damaging the slices. In this study, “Top” was defined as the estimated maximal PS amplitude obtained from the calculation of maximal PS amplitude divided by 0.9, and the value of R square was more than 0.97. “Eslope50” was the value of fEPSP slope value that resulted in half the maximal PS amplitude (half‐maximal Top value). “Hill slope” was calculated as a curve slope at “Eslope50.” Slices obtained from juvenile rats sometimes displayed small PS, almost equal to zero, or linear or parabolic E‐S curves.^19^ When E‐S coupling did not fit to a logistic curve, the values of parameters could not be calculated. Such slices were excluded from the E‐S coupling analysis.

### 2.4 Na^+^ channel α subunit and glutamate receptor GluR1 subunit gene expression

On PND 14, hippocampi were dissected from male and female offspring (seven rats each for control and 700 ppm groups). Total RNA was prepared on a FastPrep Instrument (MP Biomedicals) using the TRIzol® Reagent (Life Technologies). Reverse transcription was performed using a High Capacity RNA‐to‐cDNA Kit (Applied Biosystems Inc) in accordance with the manufacturer's protocols, and quantitative real‐time PCR was performed with real‐time TaqMan technology and a sequence detector (ABI PRISM^®^ 7000; Applied Biosystems, Inc). Gene‐specific primers for rat Na_v_1.1 (*Scn1a;* Assay ID# Rn00578439_m1; Applied Biosystems Inc), Na_v_1.3 (*Scn3a;* Rn01485335_m1), and GluR1 (*Gria1;* Rn00709588_m1), and TaqMan probes were used to analyze the transcript levels. The 18S ribosomal RNA (4319413E; Applied Biosystems Inc) served as an internal control. The reaction conditions included the initiation step for 2 min at 50°C, 10 min at 95°C, followed by 40 cycles of 15 s at 95°C (melting), and 1 min at 60°C (annealing and extension).

Rat Na_v_1.1, Na_v_1.3, and GluR1 gene‐specific primers and TaqMan probes were used to analyze transcript levels. The 18S ribosomal RNA was analyzed as an internal control and was used to normalize gene expression levels.

### 2.5 Statistical analysis

Statistical significance was evaluated using the unpaired Student's *t* test for a difference of two groups, and a repeated measure analysis of variance (ANOVA), one‐way ANOVA, Kruskal‐Wallis test for a difference among more than three groups. Spearman rank correlation coefficient was used for the analysis of statistical dependence between the rankings of two variables (age, exposure concentration) and the Steel‐Dwass test was used for post hoc analysis. The Chi‐square test of independence was used for mortality rate and the Steel test was used for post hoc analysis. For all statistical analyses, differences were considered significant at *P* < .05. Statistical tests were performed using Ekuseru‐Toukei 2010 for Windows (Social Survey Research Information Co., Ltd.).

## 3 RESULTS

### 3.1 Body weight and general growth of the offspring

Because neonatal development is known to be dependent on the physical and emotional condition of the mother,^24^ the condition of mother rats could influence both the neuronal development and the general growth of the offspring. Therefore, mortality and body weight of the newborn rats may be important indices for developmental toxicity. The number of pups that died before PND3 were significantly different between the control and 700 ppm groups (control group, 2 of 580 pups; 700 ppm group, 13 of 461 pups, *P* < .01 by the Chi‐square test for independence followed by the Steel test). Prenatal 1‐BP exposure did not show any effect on the average body weight of the offspring at PND 2 (Table 1) or of the mother rats during pregnancy and lactation periods (Figure S1 for mother rat); however, body weight gain until PND 27 was found to be inhibited in both male and female pups of the 700 ppm group (Table 1). An inhibition in body weight gain was also observed in male rats from the 400 ppm group at PNDs 7 and 14, after which, the body weight recovered to the level of the control group. There was no difference in body weight gain in females between the control and the 400 ppm group, indicating that male pups might be more sensitive to prenatal 1‐BP exposure than females. Moreover, there was no difference in the number of pups per litter between the control and 1‐BP‐exposed groups (control: 14 ± 2 heads, n = 35 litters; 400 ppm: 14 ± 3 heads, n = 5 litters; 700 ppm: 13 ± 3 heads, n = 24 litters, mean ± SD). No changes in the general development of ear unfolding, eye opening, or sexual growth, such as anogenital distance, testicular descent, and vaginal opening, was observed between the control and prenatally exposed groups (Table 2).

**Table 1:** Body weights of the offspring in the control group and the group prenatally exposed to 1‐BP

	Control		400 ppm		700 ppm	
	Mean ± SD	N	Mean ± SD	N	Mean ± SD	N
Male rats						
PND 2	7.4 ± 0.8	77	7.3 ± 0.6	25	7.2 ± 0.6	46
PND 7	17.0 ± 1.4	77	16.1 ± 1.2**	25	15.7 ± 0.9**	41
PND 14	36.1 ± 2.5	72	34.4 ± 2.9**	24	33.5 ± 1.4**	40
PND 18	47.5 ± 3.4	66	46.6 ± 2.5	20	43.9 ± 2.4**	38
PND 21	60.0 ± 4.6	66	59.5 ± 3.3	20	53.3 ± 3.7**	38
PND 27	97.4 ± 6.7	64	98.8 ± 5.5	20	86.1 ± 4.9**	38
Female rats						
PND 2	7.1 ± 0.7	62	7.1 ± 0.9	17	6.9 ± 0.7	47
PND 7	16.7 ± 1.3	62	15.8 ± 1.6	17	15.0 ± 1.3**	42
PND 14	35.3 ± 2.2	58	33.9 ± 3.1	16	31.9 ± 2.0**	37
PND 18	46.1 ± 3.0	58	45.8 ± 3.2	16	42.1 ± 2.3**	37
PND 21	58.3 ± 4.1	58	58.9 ± 4.6	16	51.9 ± 3.2**	37
PND 27	90.7 ± 6.0	57	93.5 ± 5.6	16	81.1 ± 4.9**	37

** *P* < .01 compared to the control group with one‐way ANOVA followed by Scheffe's F test.

**Table 2:** Growth milestones in control rat pups and in pups prenatally exposed to 1‐BP

	Sex	Control		1‐BP (700 ppm)	
		Mean ± SD	N^c^	Mean ± SD	N
Anogenital distance (PND 2)					
mm/g^1/3^	M^a^	1.75 ± 0.20	55	1.77 ± 0.20	49
	F^b^	0.72 ± 0.10	49	0.73 ± 0.10	44
Ear unfolding					
PND	M&F	3.2 ± 0.4	32	3.5 ± 0.5	32
Eye opening					
PND	M&F	15.6 ± 0.7	32	15.7 ± 0.8	32
Testicular descent					
PND	M	19.1 ± 0.6	16	19.1 ± 0.7	15
Vaginal opening					
PND	F	33.5 ± 0.9	28	34.0 ± 1.2	20

^a^ M represents a male rat.

^b^ F represents a female rat.

^c^ *N* represents the number of the offspring.

### 3.2 Hippocampal excitability at the age of PNDs 13 to 15

**Figure 2:**
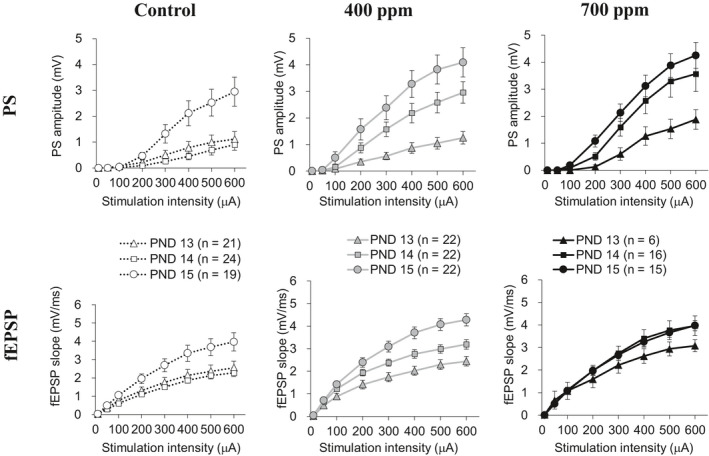
Stimulation/response (S/R) relationships of population spike amplitude (PS amplitude) and slope of field excitatory postsynaptic potential (fEPSP slope) recorded in the hippocampal CA1 region of PND 13‐15 pups from the control group and the group prenatally exposed to 1‐BP. In the control group (left panels), the S/R relationships of fEPSP slope and PS amplitude were enhanced at PND 15 (*P* < .05, repeated measure ANOVA followed by Scheffe's F test). In the 400 ppm group, the S/R relationships were enhanced 1 day earlier than the control group, at PND 14 (*P* < .05, repeated measure ANOVA followed by Scheffe's F test). In the 700 ppm group, S/R relationships of the fEPSP slope and PS amplitude indicated the similar enhancement at PNDs 14 and 15. Prenatal 1‐BP inhalation accelerated developmental enhancement of S/R relationships of fEPSP slope and PS amplitude in the hippocampal CA1 region of pups. The numbers in parentheses in the plot legends indicate the number of slices tested. Data represent mean ± standard error of mean

Developmental enhancement in hippocampal excitability was increased by prenatal VPA exposure before eye‐opening.^9^ If a given prenatally administered chemical showed the potential for inducing changes in hippocampal excitability during postnatal synaptogenesis, it would be worth investigating whether other chemicals can change excitability during the period. Here we examined whether the excitability change could be reproduced during PNDs 13‐15 in the pups prenatally exposed to 1‐BP. The S/R relationships of fEPSP slope and PS amplitude evoked in the CA1 region gradually enhanced with the increment in stimulation intensity in all three groups at PNDs 13‐15 (Figure 2). In the control pups, there were no differences in the S/R relationships of fEPSP and PS at PNDs 13 and 14, and substantial developmental enhancement was observed at PND 15. In contrast, in the 400 ppm group, the degree of enhancement of S/R relationships of fEPSP slope and PS amplitude was increased at PND 14 (*P* < .05, repeated measure ANOVA followed by Scheffe's F test), although the S/R relationships at PND 13 were similar to the control group. Furthermore, in the 700 ppm group, the enhancement of S/R relationships of fEPSP slope and PS amplitude at PND 14 was almost at the same level as those at PND 15. Finally, at PND 15, the effects of prenatal 1‐BP exposure on the S/R relationships of fEPSP slope and PS amplitude disappeared compared to those of the control group. Thus, the prenatal 1‐BP exposure enhanced the developmental changes in the hippocampal excitability, accelerating the trend at PND 14.

**Figure 3:**
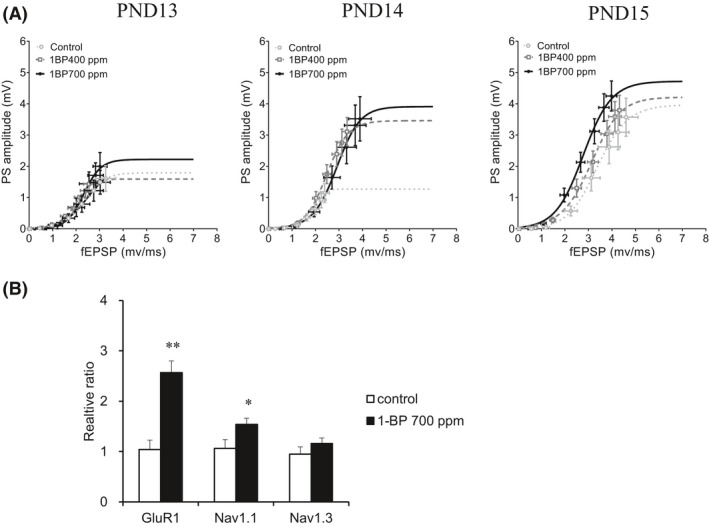
fEPSP‐Spike (E‐S) coupling curves calculated from the slope of field excitatory postsynaptic potentials (fEPSPs) and the amplitude of population spikes (PSs) and GluR1, Na_v_1.1, and Na_v_1.3 mRNA expression in the hippocampus collected at PND 14. (A) E‐S coupling curve of each group was obtained from the average of S/R relationships congregated from the slices that fitted to a logistic curve. No difference was observed among the E‐S coupling curves obtained from the control and prenatal 1‐BP‐exposed groups at PNDs 13 and 15. In contrast, E‐S coupling curves of prenatal 1‐BP‐exposed groups at PND 14 showed augmentation compared to the control group (Top value: *P* < .01, compared to the control group by Kruskal‐Wallis test followed by Steel test). See also Table 3 for other parameters and the slice number analyzed in each group. Data represent mean ± standard error of mean. (B) At PND 14, a significant increase in the mRNA expression levels of GluR1 and the Na_v_1.1 (type I) Na^+^‐channel subunit were observed in the pups prenatally exposed to 1‐BP (700 ppm) (n = 7) compared to the control (n = 7). A non‐significant increase was also observed in Na_v_1.3 (type III) mRNA expression levels. Data represent mean ± standard error of mean

Subsequently, we analyzed E‐S coupling at PNDs 13‐15. First, we fit the collected slices to a logistic curve, and their R squared values were more than 0.97. The percentages of slices that fit to a logistic curve increased during the three developmental days (*P* < .01, Spearman rank correlation coefficients followed by Steel‐Dwass test). Average percentages of slice numbers fitting to a logistic curve for three PNDs were 70%, 86%, and 90% in the control, 400 ppm, and 700 ppm groups, respectively. The percentage substantially increased in the 700 ppm group compared to the same age control group (*P* < .05, Kruskal‐Wallis test followed by Steel test). There were no differences in the E‐S coupling curves at PNDs 13 and 15 between the control and 1‐BP‐exposed groups (Figure 3A). E‐S coupling analysis determinately indicated that input‐output coupling was enhanced at PND 14 by prenatal 1‐BP inhalation. Comparison of the three parameters between the groups also showed larger values of Top and Eslope50 and smaller values of Hill slope in the 1‐BP‐exposed groups at PND 14, with the exception of Eslope50 in the 400 ppm group that showed a trend suggestive of an increase, but did not reach statistical significance (Table 3).

**Table 3:** Comparison of Top, Eslope50, and Hill slope calculated from EPSP‐Spike coupling (E‐S coupling) curves in the control, 400 ppm, and 700 ppm groups

	Control (n = 13)	400 ppm (n = 17)	700 ppm (n = 5)
	Mean ± SEM	Mean ± SEM	Mean ± SEM
PND 13			
Top	1.871 ± 0.491	1.718 ± 0.291	2.219 ± 0.493
Eslope50	2.193 ± 0.312	2.085 ± 0.201	2.293 ± 0.244
Hill Slope	1.676 ± 0.459	2.488 ± 0.596	1.100 ± 0.207

	Control (n = 16)	400 ppm (n = 19)	700 ppm (n = 14)
	Mean ± SEM	Mean ± SEM	Mean ± SEM
PND 14			
Top	1.248 ± 0.311	3.479 ± 0.510**	3.939 ± 0.786**
Eslope50	1.969 ± 0.162	2.427 ± 0.157	2.882 ± 0.308*
Hill Slope	3.608 ± 0.635	2.170 ± 0.843**	1.759 ± 0.321*

	Control (n = 15)	400 ppm (n = 21)	700 ppm (n = 15)
	Mean ± SEM	Mean ± SEM	Mean ± SEM
PND 15			
Top	3.857 ± 0.648	4.213 ± 0.530	4.925 ± 0.535
Eslope50	3.340 ± 0.283	3.173 ± 0.173	2.819 ± 0.139
Hill Slope	1.244 ± 0.179	1.259 ± 0.276	0.900 ± 0.124

Note

Numbers in parentheses are slice numbers tested. *P < .05, **P < .01, compared to the control group by Kruskal‐Wallis test followed by Steel test. Abbreviations: PND, postnatal day, Top, maximal value of PS amplitude evoked stimulation intensity divided by 0.9, Eslope50, slope value of fEPSP when PS amplitude is a half of Top value, Hill slope, slope of fitting curve at Eslope50. See also Figure 1C.

The GluR1 is a subunit of the amino‐3‐hydroxy‐5‐methyl‐4‐isoxazolepropionic acid (AMPA)‐type glutamate receptor and is involved in the generation of fEPSP slope and synaptic plasticity.^25^ Voltage‐gated Na^+^ channels are associated with the generation of action potential, which is recorded extracellularly as a PS.^26^ Based on the larger values of Top and Eslope50 observed in the 700 ppm group, the gene expression levels of these two proteins were investigated in the PND 14 hippocampus. The mRNA expression level of GluR1 was increased by 160% in the exposed group (Figure 3B). In contrast, around PND 14 there is a shift from the embryonic type (Na_v_1.3) Na^+^ channel subunits to the adult type (Na_v_1.1) subunits.^27^ Thus, we examined the mRNA expression levels of Na_v_1.1 and Na_v_1.3 and found that Na_v_1.1 expression was significantly increased (45%) in the 700 ppm group, while no significant difference was observed in Na_v_1.3 expression between the control and exposed groups.

## 4 DISCUSSION

Previously, we have evaluated the developmental neurotoxicity of chemicals before the appearance of any adverse effects in the adolescent period using an electrophysiological approach in the hippocampal slices during synaptogenesis.^9^ Here we aimed to elucidate whether prenatal exposure to industrial chemical 1‐BP changes the hippocampal excitability during synaptogenesis at PNDs 13‐15, as observed with VPA.^9^ We observed that prenatal exposure to 1‐BP vapor augmented the developmental enhancement of neuronal excitability in the hippocampal CA1 region at PND 14, immediately prior to the eye‐opening day. The increase was associated with an elevated expression of the GluR1 AMPA receptor subunit and adult type Na_v_1.1 Na^+^ channel subunit. The established adverse effect induced by prenatal 1‐BP exposure is disinhibition in the hippocampus after sexual maturation.^8^ Taken together with the present results, 1‐BP may pose a potential risk of DNT. If development background disturbances during lactation period are linked to predisposition of epilepsy, 1‐BP‐induced increase in neuronal excitability is thus an adverse effect. Enhancement of neuronal excitability was demonstrated in VPA animal model for human autistic spectrum disorder (ASD).^9^ In fact, VPA administration during pregnancy increased the risk of ASD.^28^

The ACGIH recommends an exposure limit of 0.1 ppm 1‐BP as an 8‐hour time‐weighted average, protecting against the potential for neurotoxicity, hepatotoxicity, reproductive toxicity, and developmental toxicity.^2^ The Japan Society for Occupational Health recommends an occupational exposure limit of 0.5 ppm.^29^ In the present study, considering three factors (ie, difference in species, LOAEL of 400 ppm, and developmental neurotoxicity), the exposure limit was calculated as 0.4 ppm using default uncertainty factor. However, we did not observe an effect greater than that of previous studies. Further studies investigating DNT using different approaches are needed for generalization to human risk assessment.

Maternal behaviors in the 400 ppm group at PND 1, where the mother approaches the litter, gathers the pups, licks and grooms her pups, and nurses them, seemed to be normal as observed in the control dams in our study. Mother rats in the 700 ppm group exhibited difficulties in delivery and showed high mortality, although 1‐BP exposure during pregnancy did not affect the body weight of the mother during pregnancy and lactation periods compared to the control group. Inhibition in the dam body weight gain has been reported in a different study at 100‐200 ppm inhalation (6 h/d, GDs 6‐19).^1^ We believe this difference in the observation could be due to the use of stainless‐steel cages during inhalation in the previous study, and not plastic cages with chips. Furthermore, although the body weight of the pups at PND 2 was not different between the exposed and control groups, the increase in body weight was inhibited by approximately 8%‐12% from PND 7 to PND 27 in both male and female offspring in the 700 ppm group. In the 400 ppm group, while temporal inhibition of body weight gain in male pups observed at PNDs 7 and 14 later recovered to the control level, there was no difference in the developmental increase of female body weight compared to the control group. 1‐BP is metabolized by the hepatic enzymes and is excreted in urine after conjugation with glutathione in rats.^30^ However, metabolites responsible for the inhibition of body weight gain after PND 2 have not been determined yet.

Despite substantial inhibition of body weight gain, general growth indices tested in this study were not found to be affected by the prenatal exposure. Taken together, the results indicate that prenatal exposure to 1‐BP (400 ppm, 6 h/d) for 20 days did not result in serious developmental impairment of general appearance and sexual indices. However, this does not necessarily mean that neurological functions in these pups were not influenced by prenatal exposure to 1‐BP. In fact, it was observed that prenatal exposure affected synaptic efficacy and excitability of neuronal population in the hippocampal CA1 region in the juvenile offspring. The evoked responses observed in the pup hippocampal slices at PNDs 13‐15 represent the effects of 1‐BP inhalation on developmental alteration of intrinsic neuronal excitability.

The fEPSP slope recorded from the synaptic area is interpreted as synaptic efficacy. Increase in Eslope50 upon prenatal 1‐BP exposure at PND 14 may be related to the increase in gene expression of the GluR1 AMPA receptor subunit. The postnatal weeks tested in this study include the developmental period in which the Na^+^ channel subtype is undergoing switching. Na_v_1.1 (type I) generally increases after birth and reaches a plateau around PND 30; therefore, it is regarded as the adult type (PND 15:50% of the maximum expression).^27^ The expression of Na_v_1.1 was found to be significantly increased in the group prenatally exposed to 1‐BP (700 ppm) at PND 14. Thus, increased gene expression levels of the Na_v_1.1 Na^+^ channel subunit and the GluR1 AMPA receptor subunit may be related to the increase in PS amplitude at PND 14. However, it is necessary to clarify the underlying molecular mechanisms underlying increased gene expression.

Exposure to a xenobiotic chemical during gestation may result in an increased risk of altered developmental timing. Prenatal 1‐BP inhalation and prenatal VPA administration have been shown to accelerate developmental timing of enhancement of the hippocampal excitability before the eye‐opening period. In male pups in both studies, developmental enhancement of synaptic input was accelerated, indicating an effect of DNT during synaptogenesis. Moreover, developmental increase in the PS amplitude observed around the same time could have been caused by a decrease in the tonic inhibition in the VPA study, or could be associated with an early switch to adult subtype Na_v_1.1 in the 1‐BP study. VPA was administered once at GD15, while 1‐BP in this study was inhaled for 20 days during pregnancy. Although the exposure conditions of these chemicals were different, our results suggest that the synaptogenetic period in male rats could be an adequate period to evaluate DNT of chemicals.

In the current study, we focused on developmental changes of the hippocampal excitability in male rats to compare our results obtained from VPA administration with 1‐BP exposure. However, in the PCR analysis, we included the female pups to determine whether there were sex differences in gene expression. Animal studies have demonstrated different results regarding sex differences between rats and mice. No sex differences were reported in the levels of estradiol, testosterone, and dihydrotestosterone in the rat hippocampus during the second week of age.^31^ In C57BL mice, increased expression of steroid receptor co activator‐1, synaptophysin, and GluR1 was observed at PND 14.^32^ Thus, sex differences in gene expression of GluR1 and Na^+^ channel are inconclusive and should be further investigated. Sex‐specific health effects caused by pre‐ and/or postnatal exposure/s have been reviewed for five metals (mercury, lead, manganese, cadmium, and arsenic), and are known collectively as DNT in humans.^33^ The review summarized that lead seemed to affect boys more than girls, while mercury exposure did not elucidate a clear pattern regarding sex differences in neurotoxicity. Therefore, additional research is highly warranted, especially in animal studies, where both sexes are available for testing and statistical analysis may increase the power to determine sex‐related differences in vulnerability of an immature brain to environmental/industrial chemicals.

Furthermore, to establish the experimental protocol used in our two studies as an evaluation method for chemical‐induced DNT, other known toxicants should be investigated. Studies based on substances with different chemical structures, uses, and toxic mechanisms in adult animals, including an environmental pollutant (tributyltin), an agricultural chemical (organophosphorus insecticide), and an alkene monomer (acrylamide) are underway to determine whether developmental alterations during lactation can be detected using the current experimental approach.

1‐BP is an alkyl halide, while acrylamide is type‐2 alkene. Both chemicals are classified as soft electrophiles and share similar reaction mechanisms; for instance, neurotoxicity of both 1‐BP^34^ and acrylamide^35^ was reported to have a similar underlying mechanism in animal studies.^34^, ^35^ Whether prenatal acrylamide induced DNT remains to be fully determined, since only the toxicity to body weight increase was reproducible.^36^, ^37^ In contrast, longer exposure until weaning exhibited DNT.^38^, ^39^ In our current study, the prenatal exposure to 1‐BP induced DNT. In order to explain DNT caused by prenatal acrylamide, comparison of toxicity was conducted using the same electrophysiological methods on hippocampal slices. We think that our electrophysiological evaluation method is effective in screening for DNT. Therefore, our future studies will not only focus on the chemical structure but also chemical reaction such as soft electrophiles.

In addition, our evaluation was limited to the assessment of the effects of prenatal chemical exposure on the CA1 area of rat hippocampus. We recognize that other brain areas could be assessed for their potential contribution to the onset of DNT; however, the extracellular techniques used in this study are simple and the response of CA1 neurons can be analyzed for toxicity effects.

In conclusion, this study demonstrates that prenatal exposure to 1‐BP vapor augments developmental enhancement of neuronal excitability in the hippocampal CA1 region at PND 14, just prior to the eye‐opening day. During normal development, eye‐opening is thought of as the period during which visual information is obtained and the associated neuronal activities are developed. However, in the 1‐BP group, the neuronal activity increased without processing visual information, as if neurons seemingly enhanced their function “on their own” or “arbitrarily” without visual information. Thus, our conclusion was that an increase in neuronal excitability by exposure to 1‐BP influences the developmental enhancement of neuronal excitability. The augmentation is associated with an increased expression of the GluR1 AMPA receptor subunit and adult type Na_v_1.1 Na^+^ channel subunit. Therefore, for evaluation of DNT induced by prenatal chemicals, developmental enhancement of the hippocampal excitability during synaptogenesis should be investigated before the appearance of any adverse effects.

## CONFLICTS OF INTEREST

Authors declare no Conflict of interests for this article.

## Supporting information

Fig S1
