# Supplementary material for: Exposure to 1‐bromopropane vapors during pregnancy enhances the development of hippocampal neuronal excitability in rat pups during lactation
Source: J Occup Health. 2020 Jul 26;62(1):e12135. doi: 10.1002/1348-9585.12135 (PMC7383040; doi:10.1002/1348-9585.12135)
Supplement: Supplementary file 1 — Fig S1 [file JOH2-62-e12135-s001.pptx]

## Slide 1
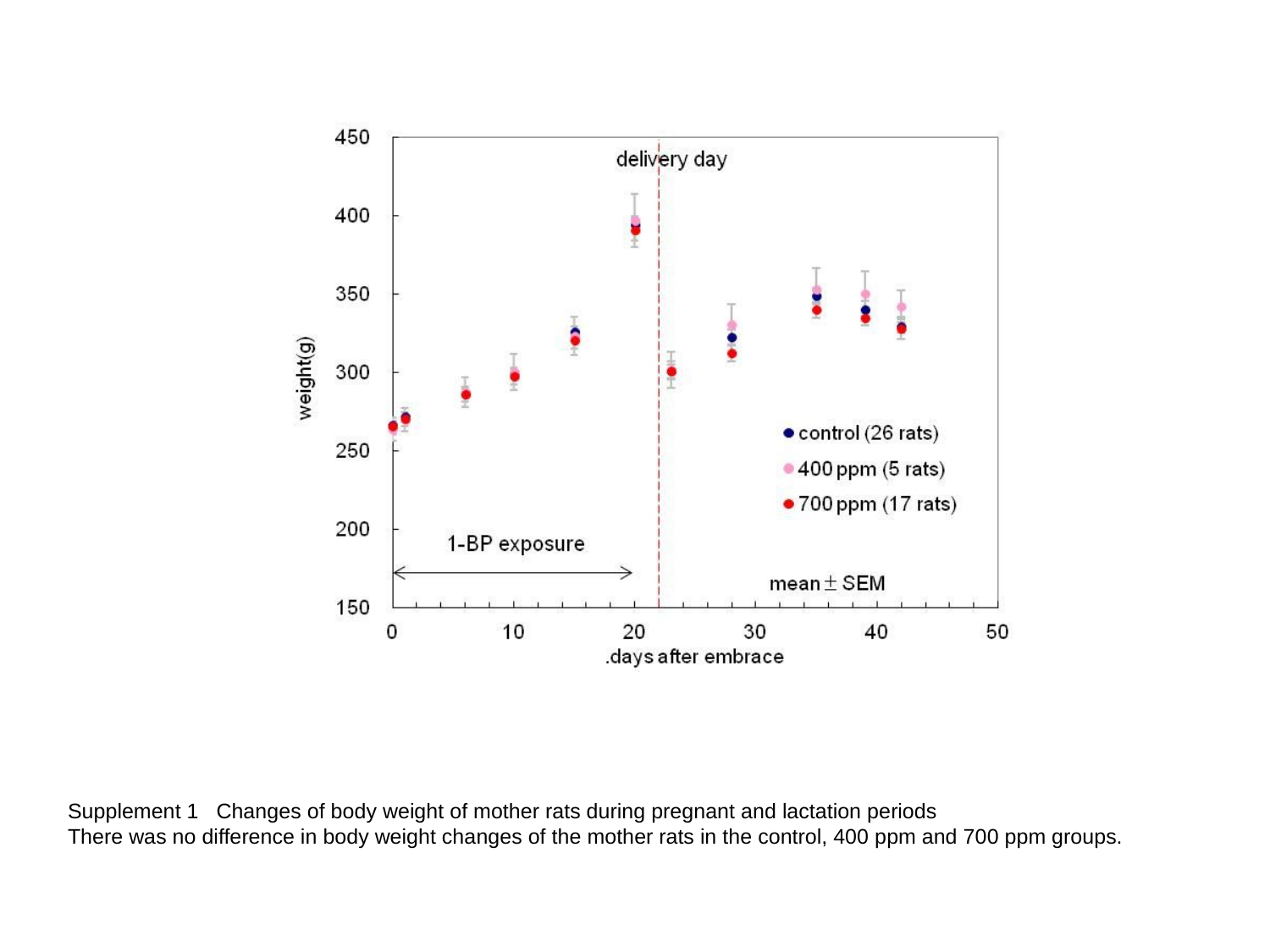

# Supplement 1 Changes of body weight of mother rats during pregnant and lactation periods There was no difference in body weight changes of the mother rats in the control, 400 ppm and 700 ppm groups.
